# Deep learning approaches for challenging species and gender identification of mosquito vectors

**DOI:** 10.1038/s41598-021-84219-4

**Published:** 2021-03-01

**Authors:** Veerayuth Kittichai, Theerakamol Pengsakul, Kemmapon Chumchuen, Yudthana Samung, Patchara Sriwichai, Natthaphop Phatthamolrat, Teerawat Tongloy, Komgrit Jaksukam, Santhad Chuwongin, Siridech Boonsang

**Affiliations:** 1 Faculty of Medicine, King Mongkut’s Institute of Technology Ladkrabang, 1 chalongkrug road, Bangkok, Thailand; 2 Faculty of Medical Technology, Prince of Songkla University, Songkhla, Thailand; 3 Epidemiology Unit, Faculty of Medicine, Prince of Songkla University, Songkhla, Thailand; 4 Faculty of Tropical Medicine, Mahidol University, Bangkok, Thailand; 5 College of Advanced Manufacturing Innovation, King Mongkut’s Institute of Technology Ladkrabang, 1 chalongkrug road, Bangkok, Thailand; 6 Department of Electrical Engineering, Faculty of Engineering, King Mongkut’s Institute of Technology Ladkrabang, 1 chalongkrug road, Bangkok, Thailand

**Keywords:** Epidemiology, Software

## Abstract

Microscopic observation of mosquito species, which is the basis of morphological identification, is a time-consuming and challenging process, particularly owing to the different skills and experience of public health personnel. We present deep learning models based on the well-known you-only-look-once (YOLO) algorithm. This model can be used to simultaneously classify and localize the images to identify the species of the gender of field-caught mosquitoes. The results indicated that the concatenated two YOLO v3 model exhibited the optimal performance in identifying the mosquitoes, as the mosquitoes were relatively small objects compared with the large proportional environment image. The robustness testing of the proposed model yielded a mean average precision and sensitivity of 99% and 92.4%, respectively. The model exhibited high performance in terms of the specificity and accuracy, with an extremely low rate of misclassification. The area under the receiver operating characteristic curve (AUC) was 0.958 ± 0.011, which further demonstrated the model accuracy. Thirteen classes were detected with an accuracy of 100% based on a confusion matrix. Nevertheless, the relatively low detection rates for the two species were likely a result of the limited number of wild-caught biological samples available. The proposed model can help establish the population densities of mosquito vectors in remote areas to predict disease outbreaks in advance.

## Introduction

The World Health Organization (WHO) has indicated that the study of entomological equipment is a key approach in combating the global outbreaks of arboviruses, which affect at least 80% of the world population at present and account for an estimated annual infection rate of 390 million^1^. The microscopic observation of mosquito vector species is the basis of entomological identification. At present, insect body landmarks and morphological keys are commonly considered as the primarily aspects to identify either vector/non-vector or local/invasive species^2–5^. The identification procedure can help evaluate the implementation of the prevention strategies based on the mosquito species, which need to be determined as either epidemic or endemic. In general, the aforementioned methods represent the routine procedures to identify insects. Moreover, the morphological identification is primarily performed by well-trained and highly skilled personnel (public health staff). This process is considered as a gold standard practice that can be replaced by automatic tools. Unfortunately, this method is time-consuming and challenging since the number of highly skilled public health personnel is inadequate and since not all of the individuals possess the required level of training, skill, and experience. In addition, these aspects may increase the cost of surveillance activities.

Alternative methods to enhance the accuracy of insect identification include intrinsically qualitative and quantitative approaches, such as polymerase chain reaction (PCR)^6^, quantitative real-time PCR^7^ and DNA barcoding^8^. Nevertheless, these methods require the utilization of expensive equipment and high-throughput technology. In addition, the methods can be performed only by highly skilled professionals with knowledge of molecular biology. In this regard, the use of automated apparatuses can help provide consistently accurate results and reduce the required amount of skilled labour.

A trap with special functionalities involving integrated feature extraction was previously constructed, which could classify small amounts of data. Nevertheless, for large amounts of data, an intelligent computer platform is required to distinguish mosquito-vectors from non-vectors based on the identification of the species through images^9^. Another system developed by using a neural network was reported to provide a high recognition accuracy (96%); however, the approach was affected by environmental variables such as the temperature, wind and insect size^10^. A trap integrated with a laser sensor and audio analysis technique was constructed by Silva and colleagues^11^, and the classification based on this approach exhibited an accuracy of up to 98% in separating the disease vectors from non-disease vectors. Furthermore, an infrared recording device exhibited an accuracy of 80% in identifying the genus and gender of *Aedes aegypti*, *Aedes albopictus* and *Culex quinquefasciatus*^12^, although the application of the developed intelligent tool was highly challenging. This challenge was attributed to the morphological characteristics of mosquitoes, which can be affected during capture or movement; moreover, managing the taxonomy was challenging, even with the assistance of a highly experienced entomologist. In another approach, the acoustic recorder within a mobile phone was used for mosquito surveillance, by sensing the insects’ sounds and mapping the distribution of the mosquito species through inexpensive tools^13^. This technique employed automatic equipment to estimate the impact of the insect infestation within an area of interest. Nevertheless, the insects’ frequency could be recorded at only small distances^13,14^, and the acquisition involved limitations pertaining to the position of the sound recorder (in front, behind, above, below, at the left or right)^15^ and period during which the insect was seeking food.

Therefore, advanced alternatives must be developed to address the challenge of identifying and quantifying mosquito vectors. In the analysis of the morphological taxonomy through image visualization, as part of the effort to develop suitable equipment, machine learning algorithms such as the support vector machine (SVM) were employed in combination with the insect morphological keys, and *Aedes aegypti* mosquitoes could be detected in photographs with an accuracy of 90%^16^. Moreover, because the approaches based on neural networks can only be applied in the case of small databases, a learning machine was developed, which could realize detection by considering the characteristic wing-shape and principal wing components of the insects. Although this method could achieve an accuracy rate of 85–100%, its application is highly complex and time-consuming, especially in the preparation of a single wing. Furthermore, specially trained and highly skilled personnel are required to obtain the detailed measurements of the wings of each acquired mosquito sample^17^.

Deep learning algorithms such as convolutional neural networks (CNNs) have been noted to be effective in several object detection and image recognition applications. For instance, CNNs have been extensively applied to detect cyclists on roads and pests in agriculture, as well as in many medical applications^18–22^. These examples indicate that CNNs represent a promising tool to be applied in the entomological field to detect the species and gender of mosquito vectors. Recently, a high-performance CNN was used to identify *Aedes* larvae by using images captured on a mobile phone^23^. However, the system produced a misclassification rate of over 30% in identifying the mosquito larvae species. Moreover, in a research based on the GoogleNet neural network, *Aedes* and *Culex* mosquitoes could be detected with an accuracy of 57–93%, and the frequency of the mosquitoes’ wing beats was investigated, which is an essential feature in classifying the species and gender. Furthermore, the developed system monitored the transfer of the pesticides or biological agents to the breeding sites to eliminate the sibling insects^15,24–26^.

Recently, Redmon and Farhadi proposed the you-only-look-once (YOLO) model^27^, which represents an object detection algorithm with a high speed and simple structure. The YOLO algorithm processes the object detection problem as a regression problem and can instantaneously predict the classification probability from the input image by using only one CNN. This detection and recognition model based on regression can instantly learn the global information of the images for end-to-end training, thereby considerably enhancing the speed of target detection. The YOLO algorithm has been noted to exhibit a high performance in terms of the accuracy and computational speed^21,27,28^. Additionally, enhanced versions of the network algorithm have been noted to operate faster than other detection frameworks.

To ensure high quality dataset collection, the photographing conditions must be controlled and standardized. Typically, the lab-acquisition protocol is applied to ensure that the contactless samples exhibit a constrained pose^29^. Moreover, although the validation of a deep learning model with field-based images is critical, it is challenging under unstandardized conditions. Since the YOLO algorithm can robustly manage both freezing images and real-time input signals, the model can be applied to solve the aforementioned problem of uncontrolled movement and/or features of living insects.

In this work, the initial- and modified-learning methods, namely, one-stage and two-stage or concatenated approaches of the YOLO model, were employed, which can be used to localize and classify a small area of a single mosquito in a complete image obtained from field-caught samples. The proposed method is considered to be a practical and effective means to identify the mosquito vector species and gender. The automatic classification of both the species and gender is crucial to establish a promising strategy for pest control. In particular, the identification of the different genders and sex ratios is a key objective in controlling the vector population density in the event of increased arthropod-borne disease occurrences^30^. Although an individual can classify the mosquito gender in a relatively straightforward manner, automatic tools can help members of the local community perform precise inspection in the case of large scale spreading. This framework can enable residents to monitor the distribution of the vector mosquitoes in their area.

The objectives of the present research were (1) to develop a platform involving a deep-learning approach to identify the main mosquito vectors and to detect annotated colour images at three different resolutions, and (2) to evaluate the performance of the platform in tests involving wild-caught mosquitoes. The model was trained and tested using a processed sort of the image quality corresponding to the specimen images by using a digital microscope, which were verified by entomologists as well. The model can be used as a deep learning prototype for mosquito identification and can likely help identify other medical vectors. Moreover, the proposed model can enable local vector surveillance and analysis and support effective vector control programmes, particularly in remote areas with an insufficient number of expert entomologists.

## Methods

### Ethics statement

This study was approved by the Animal Research Ethics Committee, King Mongkut’s Institute of Technology Ladkrabang with ACUC-KMITL-RES/2020/002. The field sampling data were obtained without harming any animal species.

### Sample collection and preparation

A total of 1585 biological samples were obtained, which included 200 specimens of laboratory strains, 1295 field-caught specimens (caught during May–July 2019) and 90 specimens of non-mosquito strains (Table 1). The model performance was evaluated, in preliminary work to determine whether the models could detect and identifying small objects, and in real situations by using the aforementioned samples that consisted of insects aged 3–5 days with intact morphological characteristics. The field-caught samples contained many dengue vectors that are extensively distributed throughout Thailand, especially during the rainy season.

**Table 1:** Biological samples.

Animal species	Gender	Sample size	Relating diseases	Collection site
*Aedes aegypti*	♀	442	Dengue, Zika, Chikungunya and Yellow fever	Kalasin, Chonburi, Bangkok and Prachuap Khiri Khan
*Aedes aegypti*	♂	373	–	Kalasin, Chonburi, Bangkok and Prachuap Khiri Khan
*Aedes albopictus*	♀	50	Dengue fever, La Crosse encephalitis, Zika, and West Nileα	Laboratory
*Aedes albopictus*	♂	50	–	Laboratory
*Aedes albopictus*	♀	2	Dengue fever, La Crosse encephalitis, Zika, and West Nileα	Chonburi
*Armigeres subalbatus*	♀	23	Brugia pahangi filariasis (Zoonotic infections)β	Chonburi
*Anopheles dirus*	♀	50	Human malariaα	Laboratory
*Anopheles dirus*	♂	50	–	Laboratory
*Culex quinquefasciatus*	♀	94	Lymphatic filariasis, St. Louis encephalitis and West Nileα	Chonburi and Bangkok
*Culex quinquefasciatus*	♂	50	–	Laboratory
*Culex spp*	♂	52	–	Chonburi
*Culex gelidus*	♀	85	Japanese encephalitis	Chonburi and Bangkok
*Culex vishnui*	♀	52	Japanese encephalitis	Bangkok
*Mansonia annulifera*	♀	14	Brugian filariasisɤ	Chonburi
*Mansonia uniformis*	♀	5	Human lymphatic filariasisδ	Chonburi
*Mansonia indiana*	♀	103	Human lymphatic filariasisɛ	Chonburi
*Musca domestica*^‡^	–	30	NA	Bangkok
*Trigona apicalis*^‡^	–	30	NA	Bangkok
*Oryzaephilus surinamensis*^‡^	–	30	NA	Bangkok
Total		1585		

^‡^ Non-mosquitoes.

When training the model during the pilot study, the specimens were obtained from the insectary of the Department of Entomology, Faculty of Tropical Medicine, Mahidol University. The insects were reared under standard conditions, fed with 5% sucrose solution ad libitum and maintained under a 12/12 h light–dark cycle at a temperature and humidity of 27 ± 2 °C and 70–80%, respectively. An expert entomologist identified the mosquito species by using the standard key taxonomy^2,3^. One hundred mosquitoes (50 male and 50 female) each of the four species were used, with *Aedes aegypti* and *Ae. albopictus* corresponding to the main and secondary dengue mosquito vectors; *Culex quinquefasciatus* and *Anopheles dirus* were the filarial and malarial vectors, respectively (Table 1).

Additionally, 90 samples of non-mosquito species were used to train the deep learning approach, consisting of *Musca domestica* (house fly), *Trigona apicalis* (stingless bees), and *Oryzaephilus surinamensis* (sawtoothed grain beetle). In general, the non-vector insect species images in a dataset are essential in the training process of a deep learning model. The non-vector images can help improve the well-trained models to distinguish between the vector and non-vector mosquitoes. In this study, we designed the experimental procedure such that the trained models could perfectly classify the non-vector species with an accuracy of 100% in the model validation process before utilizing the models to detect the objects of the vector species (Suppl. Fig S1 and Suppl. Table S2).

**Figure 1:**
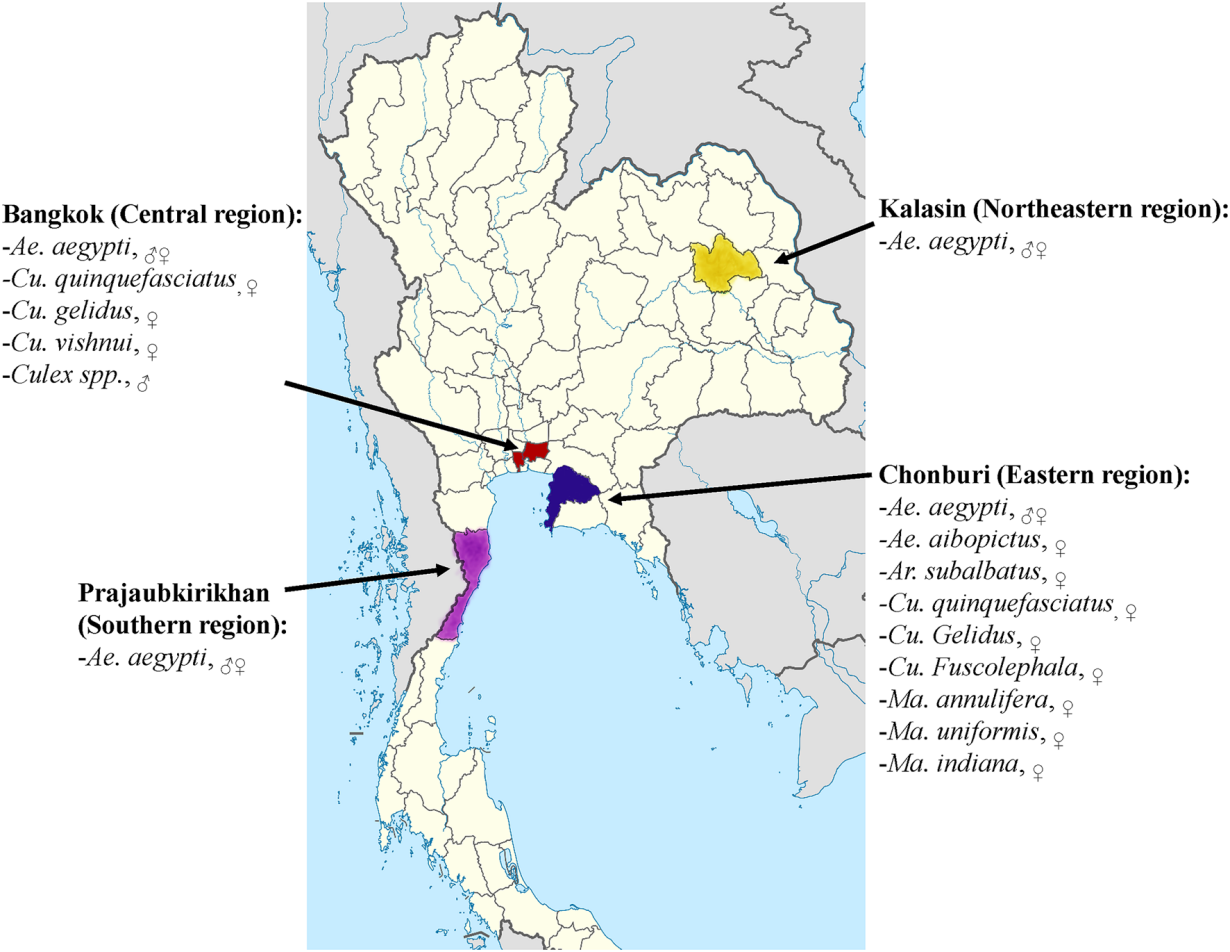
Study sites used for the sample collection. The map was originally obtained from https://upload.wikimedia.org/wikipedia/commons/a/ab/Thailand_Bangkok_locator_map.svg with license https://creativecommons.org/licenses/by/3.0/deed.en and was modified by using free version of Adobe Illustrator CC 2017 software.

To determine whether the training approach could be used to identify the samples in real settings, wild-caught samples were obtained. BG-Sentinel traps were set to capture the insects, which were later stored at − 20 °C until identification. The samples were obtained from four locations in all regions of Thailand, in which dengue was endemic, including the Kalasin Province (16°25′58.12"N/103°30′23.69"E; Northeastern), Bangkok (13° 44′ 12.1812″ N/100° 31′ 23.4696″ E; Central), Chonburi Province (13° 21′ 40.1148″ N/100° 59′ 4.8228″ E; Eastern) and Prachuap Khiri Khan Province (11°49′15.53″N/ 99°47′2.76″E; Southern) (Fig. 1).

### Dataset preparation

The anaesthetized/dead mosquitoes collected from the traps were identified. The sample was directly placed on an A4 paper sheet using a non-complex method. A non-mount slide and medium were used to prepare the sample before capturing the images. Predefined camera views of a sample were captured from dried/anaesthetized mosquitoes placed in a certain pose. The samples were individually prepared, and two-lateral, dorsal and ventral views of the samples were photographed. The mosquito images or video clips were captured using a 600× digital microscope. The captured images were obtained by cropping only the image area fitted to the individual mosquitoes. Furthermore, an expert entomologist identified each mosquito image by using the morphological landmarks of the mosquitoes through a standard stereomicroscope. The original resolution of the captured images, 1920 × 1080 pixels, was used to prepare the ground-truth labels.

**Figure 2:**
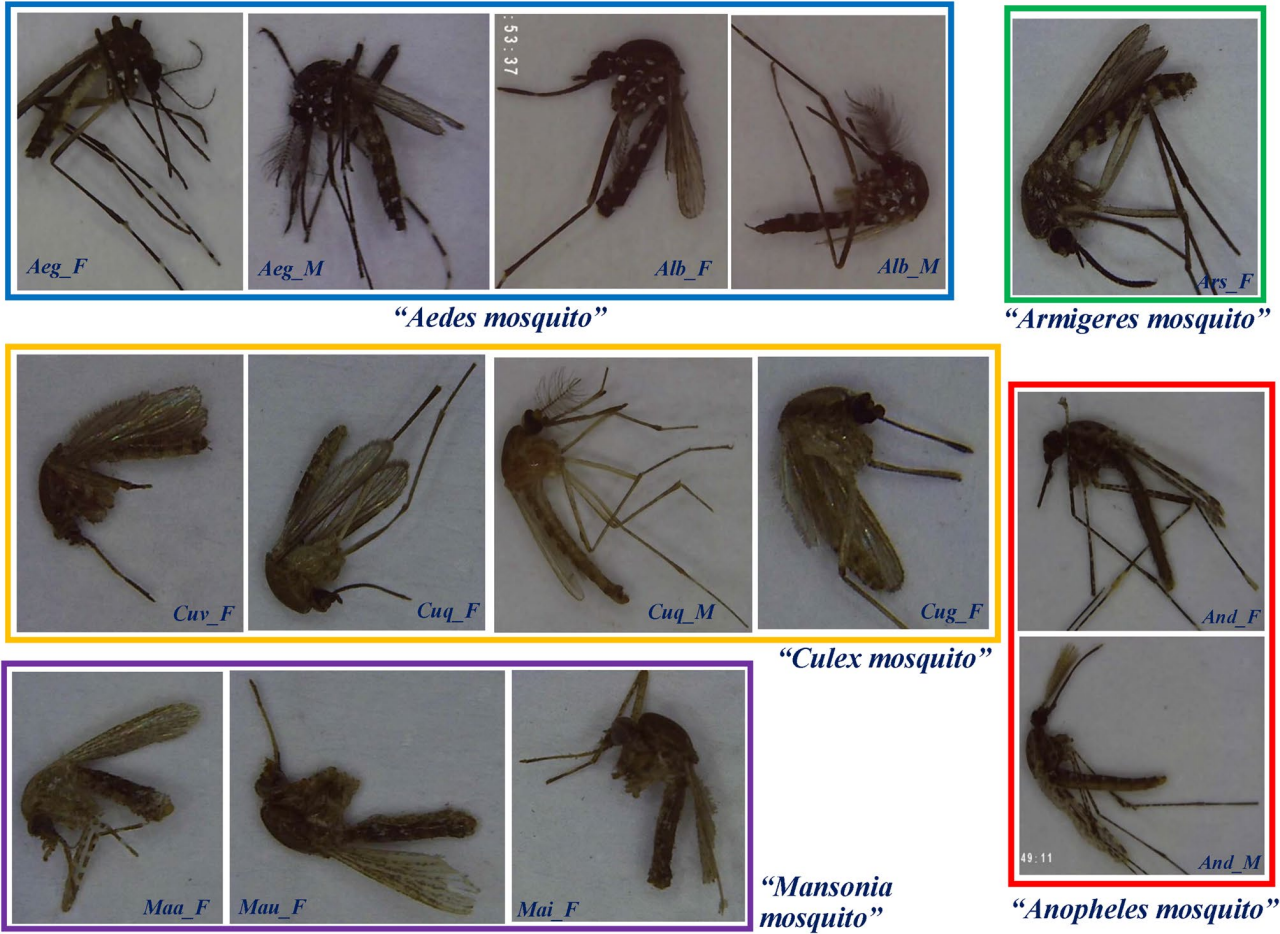
Sample categories. Wild-caught mosquitoes include Aeg_F (Ae. aegypti female), Aeg_M (Ae. aegypti male), Alb_F (Ae. albopictus female), Ars_F (Armigeres subalbatus female), And_F (An. dirus female), And_M (An. dirus male), Cuv_F (Cu. vishnui female), Cuq_F (Cu. quinquefasciatus female), Cuq_M (Cu. quinquefasciatus male), Cug_F (Cu. gelidus female), Maa_F (Mansonia annularis female), Mau_F (Ma. uniformis female) and Mai_F (Ma. indiana female). All photographs were taken by Veerayuth Kittichai, the first author in the manuscript, at Bangkok area, Thailand.

A total of 10,564 captured images were used, among which 8165 (77%) were utilized to realize the training-integrated validations and 2399 (23%) were used as the testing image sets (Suppl. Table S1). Fifteen mosquito classes were separated into different bounding boxes and labelled by professional entomologists by using the in-house deep learning software (CiRA CORE, https://www.facebook.com/groups/cira.core.comm/). The bounding boxes represented a potential region of interest (ROI) and contained images of the mosquitoes contained in the smallest area possible. The labels corresponded to the identified genus, species and gender of each mosquito and were used to train the model (Fig. 2). Overall, 190,116 augmented images based on an 8-step rotation and 9 brightness/darkness steps were used to train the model.

Subsequently, the model performance was evaluated to identify the appropriate ranges of the resolution (in pixels) as those of the input images. Three different resolutions for each category were considered: 672 × 378, 1440 × 810 and 1920 × 1080 pixels.

### Deep learning network configuration

**Figure 3:**
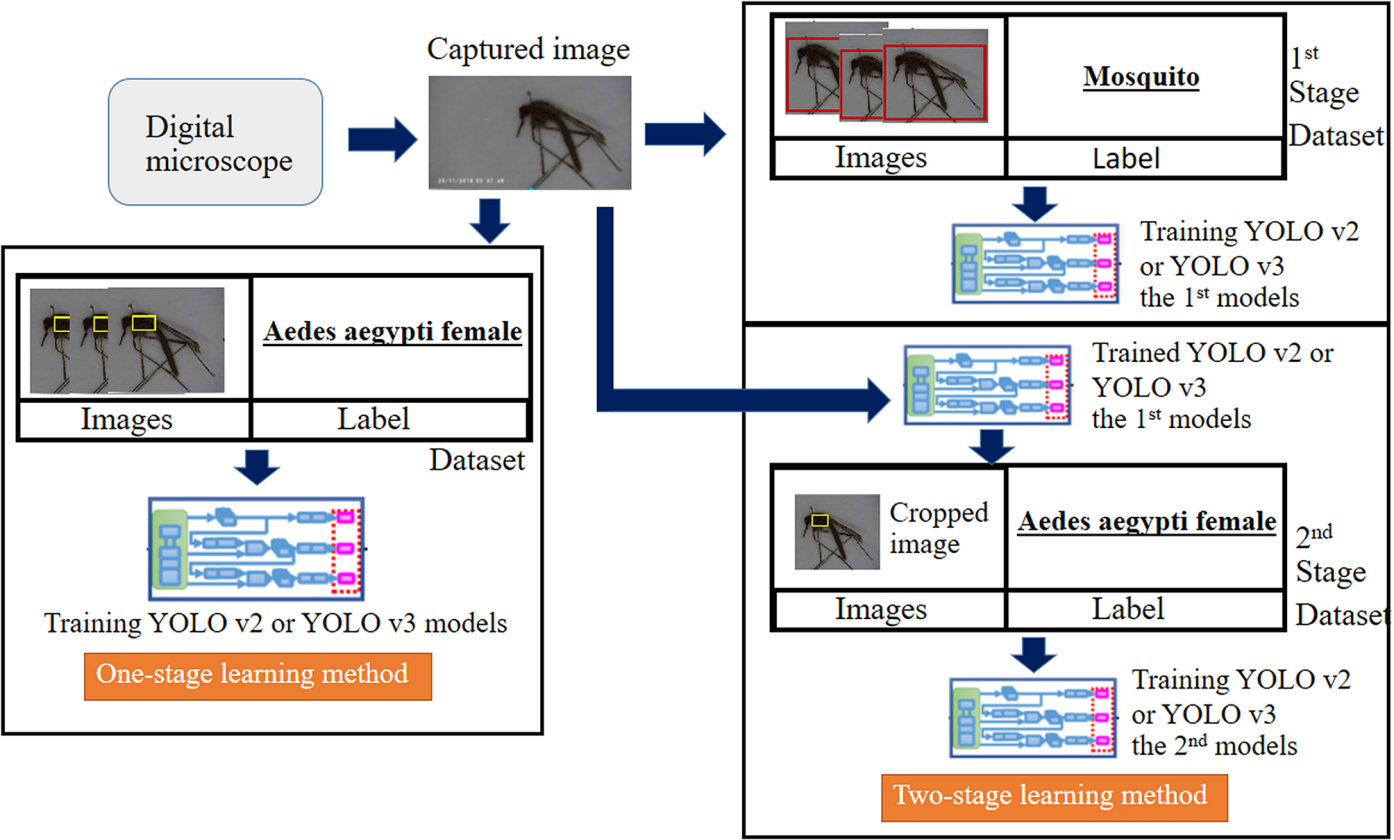

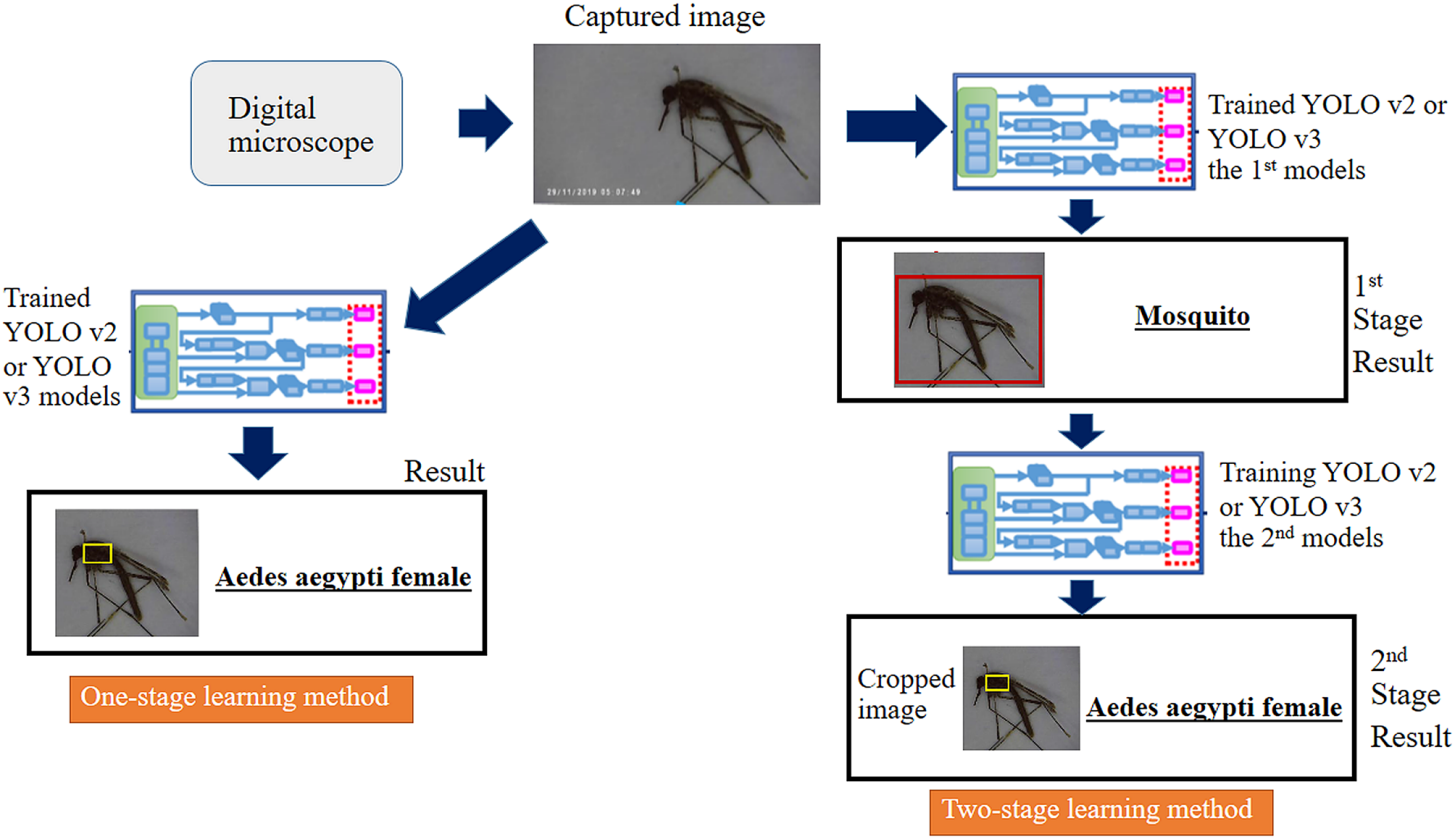
Workflow for data handling in the end-to-end neural network model, which consisted of two learning strategies, namely, the one-stage and two-stage learning methods. (1) The one-stage learning method progressed along the dark-blue dashed line, starting from the ground-truth labelling for the “genus, species and relative gender of the insect”. The ground truth labels, indicated in red rectangles, were trained in the [model] architecture. The output was displayed, pertaining to the correct relative genus, species and gender, in the output box or red rectangle, if the trained weight reached the optimal value. Under the CiRA CORE platform, the red rectangular box of the output could be selected to display or not display the value. (2) The two-stage learning method progressed along the light-blue dashed line. The start point corresponded to the ground-truth labelling for the mosquitoes and non-mosquitoes before performing the training using the [model_1] architecture, indicated in the red rectangle. The optimal trained weight was validated if could correctly distinguish between the non-mosquito and mosquito testing images. Later, the images in the set were cropped using one of the functions under the CiRA CORE programme, to be used as the dataset for the second learning process implemented using [Model_2] after labelling each cropped image pertaining to the relative genus, species and gender, as indicated in the yellow rectangle. The output could be displayed in two rectangular (red and yellow) boxes; the first box corresponded to the mosquito detection, and the second box corresponded to the classification of the relative genus, species and gender of the mosquito. Under the CiRA CORE platform, both the yellow and red rectangular boxes for the output could be selected to display or not display the values.

Collectively, six YOLO network combination models were investigated, and their performance in detecting and classifying small biological objects was assessed and compared based on the relevant learning methods. The models included the one- and two-stage methods of the tiny YOLO v2, YOLO v2, and YOLO v3 algorithms (Fig. 3). Previous studies pertaining to the characteristics of YOLO algorithms indicated the realization of prompt detection^31^. Specifically, YOLO models are prompt by design and can operate in real-time, processing 45–155 frames per second. The models can be used to detect up to 9000 categories of object classes. Additionally, the YOLO algorithm can be used to evaluate the entire area of an image with only a few false positive values after encoding the contextual information regarding the object classes^32^. A previous study reported that in cases requiring a balance of the accuracy and speed, when the training time is not restrictive, the YOLO model is suitable because the balance between the speed and accuracy is critical in practical applications^33^. Therefore, this model is suitable for realizing entomological surveillance. In this study, we adopted different versions and configurations of the YOLO algorithm to perform the identification process. The one-stage YOLO model was used to identify whether the ground-truth input contained every single-mosquito species. If every single-mosquito species was included, the image was labelled through a rectangular bounding box (the object and ROI), with the relative genus, species and gender presented above the box. In contrast, an unlabelled image was one that did not contain any mosquitoes. In the two-stage learning method (concatenated), two steps were implemented. The 1st stage of the YOLO network was used to investigate whether the whole image area contained any mosquito, and if a mosquito was detected, the cropped image of the mosquito was used for the subsequent processing. The independent YOLO network in the 2nd step was used to identify a particularly relative genus, species and gender within the cropped image derived from the image containing the mosquito, which was obtained in the first step.

For the model training process shown in Fig. 3a, the captured images were transferred to a dedicated deep learning server to realize the labelling and model training. The labelling tool available in the CiRA CORE platform was employed. Two schemes were considered to train the model, namely, one-stage and two-stage learning methods. To prepare the dataset for the one-stage learning method, the captured mosquito images were manually labelled with their corresponding species, genus and genders. Subsequently, the dataset was fed to either the YOLO v2 or YOLO v3 model to realize the learning process in the CiRA CORE platform. The learned parameters were utilized in the runtime testing in the following process.

In the two-stage learning method (Fig. 3a), the dataset was prepared in two sequential stages. The captured mosquito images were manually labelled with the label “Mosquito”. Moreover, we input non-vector insect species to the dataset since such samples are crucial to realize the distinction between the vector and non-vector mosquitoes. The non-vector insect images were labelled “Non-mosquito”. Next, the 1st stage dataset was fed to either the YOLO v2 or YOLO v3 model to implement learning process. Using the parameters learned from the 1st stage dataset, the trained model was used to prepare the cropped mosquito images by operating the trained model with the captured images as the input. One of the model outputs corresponded to the information regarding a bounding box of a mosquito object. This bounding box was used to provide the position and area for cropping the mosquito images. Subsequently, the 2nd stage dataset of the cropped mosquito images was manually labelled with the corresponding species, genus and gender of the mosquitoes. This dataset was fed to either the YOLO v2 or YOLO v3 model for the learning process. The learned parameters were later used in the runtime experiment in the subsequent process.

This study was performed using a dedicated deep learning server with a 64-bit Ubuntu 16.04 operating system with a library based on the *CUDA 10.1* toolkit and *CUDNN7.5.0*. All the experiments were conducted on the server with the following configuration: *CPU i7-8700 (3.70 GHz), RAM 16* × *2 GB, GPU NVIDIA GTX 1070Ti (8 GB),* using the *C*++ programming language. The models were fine-tuned with a total of 16 (tiny YOLO v2), 32 (YOLO v2) and 106 (YOLO v3) layers.

The training datasets consisted of 8,165 laboratory and field-caught images. The aforementioned deep learning model was implemented in the in-house deep learning software (CiRA CORE), and the same computational conditions were applied for all the models. An intersection of union (IoU) of 0.5 was used to compare the average precision for each object. A state-of-the-art network algorithm based on the YOLO and YOLO9000 frameworks was modified, as the original algorithm involved certain limitations when using the regression pipelines to detect objects. Moreover, in the model construction, a smaller run time and lower memory consumption were emphasized. A trained model with a mini batch sized 64 (min/max) and involving 16 subdivisions was used on a 1070Ti GPU, with a momentum and decay of 0.9 and 0.0005, respectively. The learning rate was adjusted as 0.001 with a burn, step value, and learning scale rate of 1000, (400,000, 450,000) and (0.1, 0.1), respectively. Additionally, the two-stage learning method of the YOLO v3 was adopted and compared to the previously applied one-stage method. This two-stage method combined both object localization and simulation classification techniques. Hence, an objective of this study was to examine whether the two-stage YOLO method exhibits notable advantages in the detection process, in terms of both the speed and accuracy in cases involving images of dense objects.

Figure 3b illustrates the model testing process. In the one-stage leaning method, the captured images were fed directly to the trained model. The model output corresponded to the genus, species and gender of the mosquitoes. In the two-stage learning method, the captured images were fed through the 1st and 2nd stage models consecutively.

### Classification of application input

The performance of the deep learning network models was assessed using confusion metrics. Several statistical variables from these models were compared, including the precision, recall (sensitivity), accuracy^31^, F1 score, misclassification rate and specificity. The formulas for these parameters are as follows:

1 $$Precision= \frac{Tp}{Tp+Fp}$$

2 $$Recall= \frac{Tp}{Tp+Fn}$$

3 $$Accuracy=\frac{Tp+Tn}{Tp+Fp+Tn+Fn}$$

4 $$Specificity=\frac{Tn}{Fp+Tn}$$

5 $$Misclassification \;rate=\frac{Fp+Fn}{Actual \;positive+Actual \;negative}$$

6 $$F1 \;score=\frac{2\times Precision\times Recall}{Precision+Recall}$$

where *Tp, Tn, Fp,* and *Fn* denote the number of true positive, true negatives, false positive and false negatives, respectively. The actual positive and actual negative represent the sum of the number of true positive and false negative and sum of the number of true negatives and false positives, respectively.

In addition, a confusion matrix table was constructed to realize a class-wise comparison to examine the accuracy of the identifications (generalized accuracy) and ensure that the model could detect and classify objects in a reliable manner. The mean accuracy determined from the table was analysed and assessed for all the models.

The receiver operating characteristic curve (ROC) was constructed with a 95% confidence interval, and the area under the curve (AUC) was established to assess the model accuracy. The ROC analysis was performed using the SPSS software.

## Results

This performance of six modifications of the YOLO algorithm, implemented within the Darknet-19 and Darknet-53 backbones was evaluated. The objective was to determine the optimal learning strategy and neural network model of the YOLO algorithm versions to be employed in the entomological field to identify the main mosquito vectors and overcome the limitations of the models in examining the images of wild-caught mosquitoes under different conditions.

### Assessing the robustness of the proposed networks

The performance and robustness of the proposed model were tested using the in-house deep learning software (CiRA CORE). The video of the performance evaluation process is illustrated in Supplementary Table S1 and hosted on YouTube at https://youtu.be/7yI5C3h5yYk. In the initial and modified learning processes of the YOLO models, single-mosquito images were used in the training and testing sets, as shown in Table 1 and Fig. 2, and the different network architectures of the models were assessed considering the same assignments. The statistical results indicated that the accuracy of the YOLO v3 model was higher than that of the other models (Table 2). Nevertheless, for each model, the modified two-stage learning method outperformed the one-stage YOLO model. The mean average precision (mAP) and adjusted mAP ranged from 73.80–85.9% and 87.2–99.0%, respectively, as indicated in Table 2. Since only 15 classes of mosquito vectors were employed in this study, the results obtained through the single-class predictions of this model can be considered to be encouraging for the application of deep learning models in mosquito-vector identification. In addition, the recall (sensitivity) values indicate that the models exhibited a high sensitivity ranging from 70.8 to 92.4% with adjusted values of 88.5–88.9%. Both the specificity and accuracy were highly reliable, ranging from 98.4–99.4% and 97.0–98.9%, respectively. The results indicated low misclassification rates, ranging from 1.1 to 3%. Moreover, a high F1 score was obtained, and the harmonic mean for the precision and recall ranged from 70.6–85.3% and from 81.0–88.9%, respectively, after adjustment. The overall results indicated that the two-stage YOLO v3 network model outperformed all the other models in the identification of entomological images of small objects in a large environment.

**Table 2:** Model-wise identification performance of the YOLO networks.

Model	Mean average precision	Recall	Specificity	Accuracy	Misclassification rate	F1 score
One-stage tiny YOLO v2	0.730 ± 0.178 *[0.839 ± 0.093]*	0.708 ± 0.168 *[0.810 ± 0.092]*	0.984 ± 0.010	0.970 ± 0.011	0.030 ± 0.011	0.706 ± 0.165 *[0.810 ± 0.075]*
Two-stage tiny YOLO v2	0.832 ± 0.126 *[0.913 ± 0.049]*	0.864 ± 0.063	0.990 ± 0.007	0.980 ± 0.008	0.020 ± 0.008	0.828 ± 0.096 *[0.888 ± 0.043]*
One-stage YOLOv2	0.798 ± 0.183 *[0.921 ± 0.043]*	0.756 ± 0.175 *[0.865 ± 0.083]*	0.991 ± 0.006	0.983 ± 0.006	0.017 ± 0.006	0.765 ± 0.177 *[0.882 ± 0.060]*
Two-stage YOLO v2	0.820 ± 0.151 *[0.990 ± 0.069]*	0.812 ± 0.140 *[0.870 ± 0.070]*	0.992 ± 0.005	0.986 ± 0.007	0.014 ± 0.007	0.810 ± 0.144 *[0.868 ± 0.078]*
One-stage YOLO v3	0.826 ± 0.167 *[0.928 ± 0.044]*	0.834 ± 0.146 *[0.889 ± 0.071]*	**0.994 ± 0.005**	**0.989 ± 0.005**	**0.011 ± 0.005**	0.818 ± 0.157 *[0.873 ± 0.049]*
Two-stage YOLO v3	**0.859 ± 0.154** *[0.956 ± 0.020]*	**0.924 ± 0.053**	**0.994 ± 0.005**	**0.989 ± 0.006**	0.012 ± 0.006	**0.853 ± 0.128** *[0.889 ± 0.035]*

Performance metrics analyzed represent the value and its 95% confidence interval. The adjusted values [italics characters in brackets] were calculated when the response value was less than 50%; this may bias the results toward the classes with small values.

Bold value represents the highest value of the proposed statistical parameters by model.

**Figure 4:**
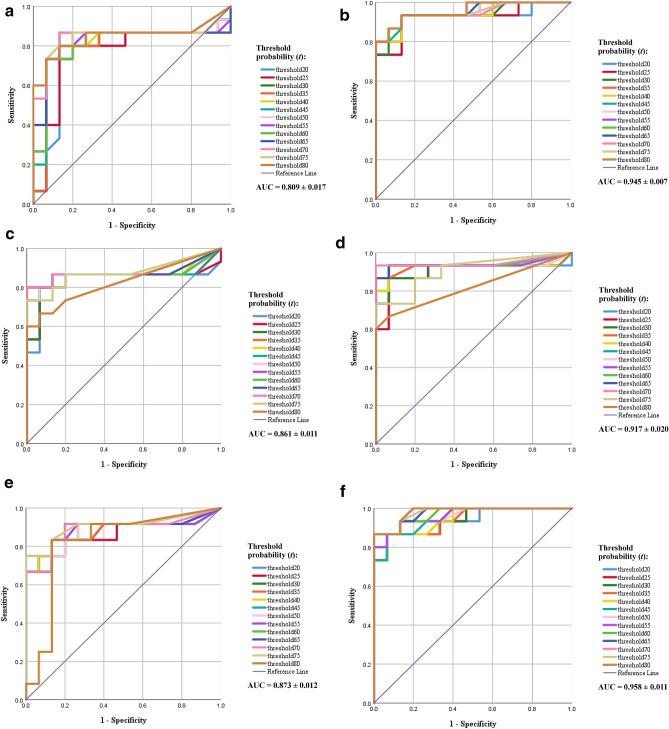
ROC curve and average AUC for each model and the threshold probability of the learning method with the YOLO network models. (**a**,**b**) Correspond to the one-stage and two-stage methods of tiny YOLO v2, respectively. (**c**,**d**) Correspond to the one-stage and two-stage methods of YOLO v2, respectively. (**e**,**f**) correspond to the one-stage and two-stage methods of YOLO v3, respectively.

The robustness of the models was evaluated and determined considering a threshold probability, P(*t*), from *t*_20%_ to *t*_80%_. The identifications resulting from each model corresponded to *P*_class_ ≥ *t*^34^. The sweeping values of *t* in the ROC ranged from 0 to 1 (Fig. 4a–f).

On average, the AUC was 0.809 ± 0.017 and 0.945 ± 0.007 for the one- and two-stage versions of tiny YOLO v2, respectively, 0.861 ± 0.011 and 0.917 ± 0.020 for the one- and two-stage versions of YOLO v2, respectively, and 0.873 ± 0.012 and 0.958 ± 0.011 for the one- and two-stage versions of YOLO v3, respectively (Table 3). The results are in agreement with the aforementioned mAP results and indicate that the two-stage learning models with the YOLO v3 network outperformed the other models in the object detection in single-mosquito images by using the general criteria of morphological taxonomic keys.

**Table 3:** Determination of AUC by threshold probability (*t*).

Threshold probability (t)	Area under curve each model					
	One-stage tiny YOLO v2	Two-stage tiny YOLO v2	One-stage YOLO v2	Two-stage YOLO v2	One-stage YOLO v3	Two-stage YOLO v3
20%	0.758	0.920	0.833	0.898	0.867	0.933
25%	0.760	0.924	0.840	0.900	0.861	0.938
30%	0.791	0.936	0.849	0.909	0.869	0.938
35%	0.796	0.942	0.867	0.922	0.864	0.940
40%	0.807	0.942	0.869	0.927	0.878	0.944
45%	0.811	0.949	0.862	0.933	0.872	0.951
50%	0.813	0.951	0.867	0.933	0.872	0.960
55%	0.809	0.956	0.871	0.938	0.881	0.962
60%	0.809	0.956	0.871	0.940	0.886	0.971
65%	0.827	0.953	0.876	0.942	0.886	0.976
70%	0.844	0.947	0.884	0.947	0.889	0.980
75%	0.847	0.949	0.876	0.907	0.906	0.978
80%	0.844	0.956	0.824	0.820	0.819	0.980
Mean ± 95% CI	0.809 (0.792, 0.826)	0.945 (0.938, 0.952)	0.861 (0.850, 0.872)	0.917 (0.897, 0.937)	0.873 (0.861, 0.885)	0.958 (0.947, 0.969)

### Multi-class detection

The class-wise performance of the trained model was assessed considering the dataset created by organizing each image in each class in a separated folder. The prediction results in terms of the mAP and adjusted version are summarized in Table 4. Almost all the classes of mosquitoes, such as the *Aedes*, *Armigeres*, *Anopheles*, *Mansonia* and *Culex* mosquitoes, exhibited high mAP values. Interestingly, the one- and two-stage YOLO v3 models could identify the *Cu.* spp. Male, *Cu. gelidus* and *Cu. quinquefasciatus* females at a rate of almost 100%. Nevertheless, the prediction results of the two species exhibited low mAP values. This aspect could be attributed to the small number of images for these species in the training set. Using a training set involving more than 300 unaugmented images could enhance the prediction values obtained using the YOLO v2 and YOLO v3 network models.

**Table 4:** Comparison of detection results using different versions of one-stage and two-stage YOLO models.

Model	mAP (adjustment)	*Ae. aegypti*	*Ae. aegypti*	*Ae. albopictus*	*Ae. albopictus*	*An. dirus*	*An. dirus*	*Ar. subalbatus*	*Ma. annulifera*	*Ma. uniformis*	*Ma. indiana*	*Cu. gelidus*	*Cu. quin*	*Cu. quin*	*Cu.* spp	*Cu. vishnui*
		♀	♂	♀	♂	♀	♂	♀	♂	♀	♂	♀	♀	♂	♀	♀
One-stage tiny YOLOv2	0.730 (0.872)	0.841	0.447	0.884	**0.993**	0.921	0.936	0.910	0.881	0.038	**0.941**	0.585	0.826	0.913	0.833	0.000
Two-stage tiny YOLOv2	0.832 (0.913)	0.890	0.698	0.896	0.956	0.985	0.990	0.871	**1.000**	0.254	0.927	0.961	0.900	0.835	0.962	0.357
One-stage YOLOv2	0.798 (0.921)	**0.998**	0.871	0.904	0.977	0.777	0.987	0.920	0.809	0.004	0.940	0.996	0.889	0.911	0.992	0.000
Two-stage YOLO v2	0.820 (0.909)	**0.998**	0.874	0.988	0.984	0.807	**0.991**	0.902	0.999	0.000	0.627	0.787	0.991	0.881	0.986	**0.484**
One-stage YOLO v3	0.826 (0.928)	0.991	0.810	0.984	0.987	0.960	0.927	**0.986**	0.797	0.223	0.923	0.841	**1.000**	0.960	**1.000**	0.000
Two-stage YOLO v3	0.859 (0.956)	0.984	0.948	**0.998**	0.974	**0.990**	0.931	0.909	0.981	0.183	0.905	**1.000**	0.939	**0.966**	**1.000**	0.176
No. training images (un-augmentation)		929	815	702	650	488	507	461	309	255	504	464	626	455	537	251

The mAP represents the mean average precision. The mAP_adj represents the adjusted mAP which was calculated without the precise values of *Ma. uniformis* and *Cu. vishinui.* The adjusted values were used to avoid bias, which may reduce the mAP. *Cu. quin* represents *Culex quinquefasciatus.*

Bold value represents the highest value of the proposed statistical parameters by model.

A confusion matrix was constructed to facilitate the evaluation and explanation of the model performance in identifying the species. According to the tables corresponding to the various YOLO models (Supplementary Tables S3.1–3.6), the proposed models could not identify *Cu. vishnui*. Moreover, *Ma. uniformis* could be detected using YOLO v3 but not YOLO v2. The results demonstrated the high quality of both the one- and two-stage learning methods with the YOLO v3 model. Due to the different numbers of specimens in each class of the training set, the overall accuracy exhibited different values of less than 90%, specifically, 73.00%, 83.20%, 83.36%, 87.99%, 86.24%, and 88.55% for the one-stage and two-stage YOLO-tiny v2 models, one-stage and two-stage YOLO v2 models, and one-stage and two-stage YOLO v3 models, respectively. Under the remaining classes, the model provided more reliable results ranging from 95 to 100% when using the YOLO v3 network models, consistent with the aforementioned mAP results.

### Pixel resolutions for the detection decision

The deep neural networks were examined to determine if the number of pixels in an image and the ability of the proposed models to identify the specimen were correlated. To examine this aspect, three different pixel densities were considered to identify a mosquito presented in images with different background intensities. The modified YOLO v3 model, which was the model with the highest performance, was used to detect the identity of a single mosquito from images with three different pixel resolutions, and the results were as presented in Table 5. The images with a higher resolution (1440 × 810 and 1920 × 1080 pixels) corresponded to better results compared to those obtained using images with a lower resolution (672 × 378 pixels). This result differed from those of a previous study, which indicated that the 608 × 608 pixel resolution image produced a lower mAP than that of images with resolutions of 416 × 416 and 320 × 320 pixels^31^. This phenomenon likely occurred because in the previous study, a well-trained model was used, and the objects to be identified (leukocytes) were presented in different surroundings in the images.

**Table 5:** Detection success based on different pixel resolutions.

Model	Resolutions	mAP	Accuracy	Sensitivity	Specificity
Two-stage YOLO v3	672 × 378	0.948	0.986	0.898	0.992
	1440 × 810	0.957	0.988	0.926	0.993
	1920 × 1080	0.956	0.989	0.924	0.994

The original images had a pixel resolution of 1920 × 1080 pixels. These were rescaled to 672 × 378 and 1920 × 1080 pixels before the comparative model testing was conducted.

## Discussion

According to the result in this study, the state-of-art models is the greatest performance when comparing to others^9,35–38^, except for the precision reported by Minakski et al. (Suppl. Table S5)^36^. The proposed model exhibited an excellent performance with high precision and recall/sensitivity levels of more than 92% in identifying the genus and species of mosquito vectors considering the morphological characteristics of the mosquitoes. Moreover, all the considered classes of mosquitoes could be detected through the various models with a high accuracy (97–98.9%) and specificity (98.4–99.4%), as indicated in Table 2. These results are comparable to those of the existing classifiers and network models^9,21,24^. Notably, the well-trained model shows the sensitivity (92.4%), the specificity (99.40%), the precision (95.56%) and the accuracy (98.9%) to detect the species and gender of mosquito vectors. Specifically, the mAP of the YOLO v2 model is 99%. Nevertheless, based on the class-wise detection results, as indicated in Table 5, the proposed models outperformed the existing models^9,39,40^. Besides, although the dataset used is unbalanced, the overall accuracy in the study shows more than 98% to 100% in identifying between the same species and gender of *Aedes aegypti*, *Aedes albopictus*, *Culex quinquefasciatus* (Suppl. Table S3.6), when comparing to that from previous studies^9,35,37^. This aspect could be attributed to the proposed models being extensively trained using a dataset that was carefully prepared using the original and largest variant in terms of both complete and incomplete morphological characteristics from the samples collected from four study sites in Thailand. Consequently, the proposed model can be used in real settings to realize vector-identification.

In general, intact mosquitoes are distinguished based on the colour and pattern of the head (proboscis and palpi), body (terga and abdomen), wing, thorax (mesonotum) and leg (femur and tarsi)^41,42^. Nevertheless, the variability of the morphological characteristics and texture of the mosquitoes may be degraded owing to discoloration caused during the capture and processing at the study site or during the freezing and drying preservation processes^35^. In this context, the YOLO model can realize the fast localization and classification of highly variable poses of preserved mosquito samples, similar to the work of non-experts, in identifying the different mosquito species.

Rationales may possibly support our empirical finding (described as above) such as sample size, ranges of image resolution and a potential hybrid model of the object detection method. Firstly, a bunch of sample size and their diversity help improve an ability of the neural network model in recognizing the pattern recognition of them^9^. Nevertheless, the classification performance of the models in identifying the particular classes of mosquitoes was strongly correlated with the number of images in each class of the mosquitoes used in the training set (Table 4). As the samples belonging to the same species or class may not be highly variable, the model performance can likely be enhanced by using an enlarged dataset involving more than 300 images for each class. In addition, the problem of the lack of variability in the mosquito images can be alleviated by using the augmentation function available in the in-house deep learning software (CiRA CORE), which provides 360° rotational angles, enhanced image contrast, and Gaussian noise and blur conditions. Although this process may introduce an additional step in the image annotation process before the training of the deep neural network, the quality of detection can be enhanced^34^. Furthermore, as reported, the proposed models cannot identify the objects when using low-quality images with a low resolution as the input^18,43^, and a low image resolution may degrade the result of the identification^44^. Suitable ranges for the image resolution should be determined to increase the detection rate of the model. In addition, the specifications of the digital cameras used to capture the images must be examined, since digital cameras can produce a large range of image resolutions, and this aspect may influence the identification performance of the proposed models. This problem can be solved by training the models using sets of images having a variety of pixel resolutions to establish the optimum resolution to train the model. In this study, this procedure was attempted to be implemented by testing one of the models with images having three different pixel resolutions. The outcome suggested that the YOLO v3 model can be applied across a wide range of pixel resolutions, as indicated in Table 5. Lastly, the hybrid approach between darknet-53 and YOLO versions model may be suitable to work with the entomological task, because it can help us find the object location and also classify a small object within a large environment each image^45^. Although a hybrid neural network model between deep learning and machine learning model is considerably efficiency, a major problem of the proposed detector found is how to select the high quality image dataset and also the strategy for labeling an appropriate ground-truth as compatible with an objective. A recent study has been proved that a renewal dataset specific to his purpose improved the performance of the proposed detector up to 81% of average precision when comparing to a former report^46^. Overall, the deep learning approach is practical and comfortably accessible for the crowed community to facilitate the prevention effort against the mosquito-borne disease.

The comparative results of the three deep learning models using one- and two-stage learning methods demonstrate that, in general, the two-stage learning method exhibits a higher identification performance than that of the one-stage method (Table 2). This study represents the first attempt to direct compare the two methods in identifying insect vectors based on image-training sets. Nevertheless, all the considered network-learning method combinations produced reliable descriptions of both intra- and inter-observed genera, even for incomplete field-caught mosquito samples. The application of the YOLO v3 model combined with the two-stage learning method corresponded to the highest detection rate, based on the morphological features of the mosquito classes included in the training set.

## Conclusion

This study used one- and two-stage learning methods of the YOLO network models as a detection algorithm to identify disease-transmitting mosquito vectors. In the one-stage method, the machine was trained using a dataset to predict the insect species. In contrast, the two-stage method (concatenated) involved model training in two rounds using the same dataset to detect the insect of interest and identify the relative species. To evaluate the model performance, the models were trained using the images to identify the features of various vector and non-vector species under various image resolutions. The training dataset involved 190,116 single images (augmented) grouped into 15 different classes to recognize the genus, species and gender of the mosquitoes. The state-of-the-art network model could effectively discriminate the species and gender of the insects when images from the testing dataset were input. The results demonstrate the ability of the proposed model in estimating the population density after identifying the various mosquitoes, many of which are vectors for arboviruses, which can cause disease outbreaks in remote areas^47^. The proposed model exhibited a high accuracy of 0.96, as indicated by the ROC and AUC analysis, and this value is higher than those reported in the literature^47–49^. Furthermore, the detection procedure was prompt, which can accelerate the species identification to instantly establish if disease-transmitting mosquito vectors are present.

The empirical results suggest that the proposed models can facilitate the detection and classification of mosquito vectors in field sites and realize rapid screening and identification to support the local public health staff. The models can further be used to evaluate the population density of mosquito vectors that may transmit mosquito-borne diseases in remote areas, by enhancing the models by introducing quantitative functions. Finally, the identification model can be extended to an android application for fieldwork. This framework can help public health workers in identifying and counting the mosquito species, thereby enabling the early detection of disease outbreaks in areas subject to periodic epidemics.

## Supplementary Information

Supplementary Information 1.

Supplementary Information 2.

Supplementary Information 3.

## Data Availability

The data supporting the findings of this study are available from the corresponding author upon reasonable request.
