# Supplementary material for: Deep learning approaches for challenging species and gender identification of mosquito vectors
Source: Sci Rep. 2021 Mar 1;11:4838. doi: 10.1038/s41598-021-84219-4 (PMC7921658; doi:10.1038/s41598-021-84219-4)
Supplement: Supplementary file 1 — Supplementary Information 1. [file 41598_2021_84219_MOESM1_ESM.docx]

List of supplementary figures

**Supplementary Figure S1. Object detection of mosquitoes (in the top and middle rows) and non-mosquitoes (in the bottom row)**. The figure shows the results of the two-stage-learning strategy based YOLO v3 model. The detection of a mosquito in the bounding box was indicated by the word “mosq” (representing mosquito), and the relative genus, species and gender of the mosquito were specified. No labelling was performed for non-mosquitoes.
