# Supplementary figures and images for: Deep learning approaches for challenging species and gender identification of mosquito vectors
Source: Sci Rep. 2021 Mar 1;11:4838. doi: 10.1038/s41598-021-84219-4 (PMC7921658; doi:10.1038/s41598-021-84219-4)

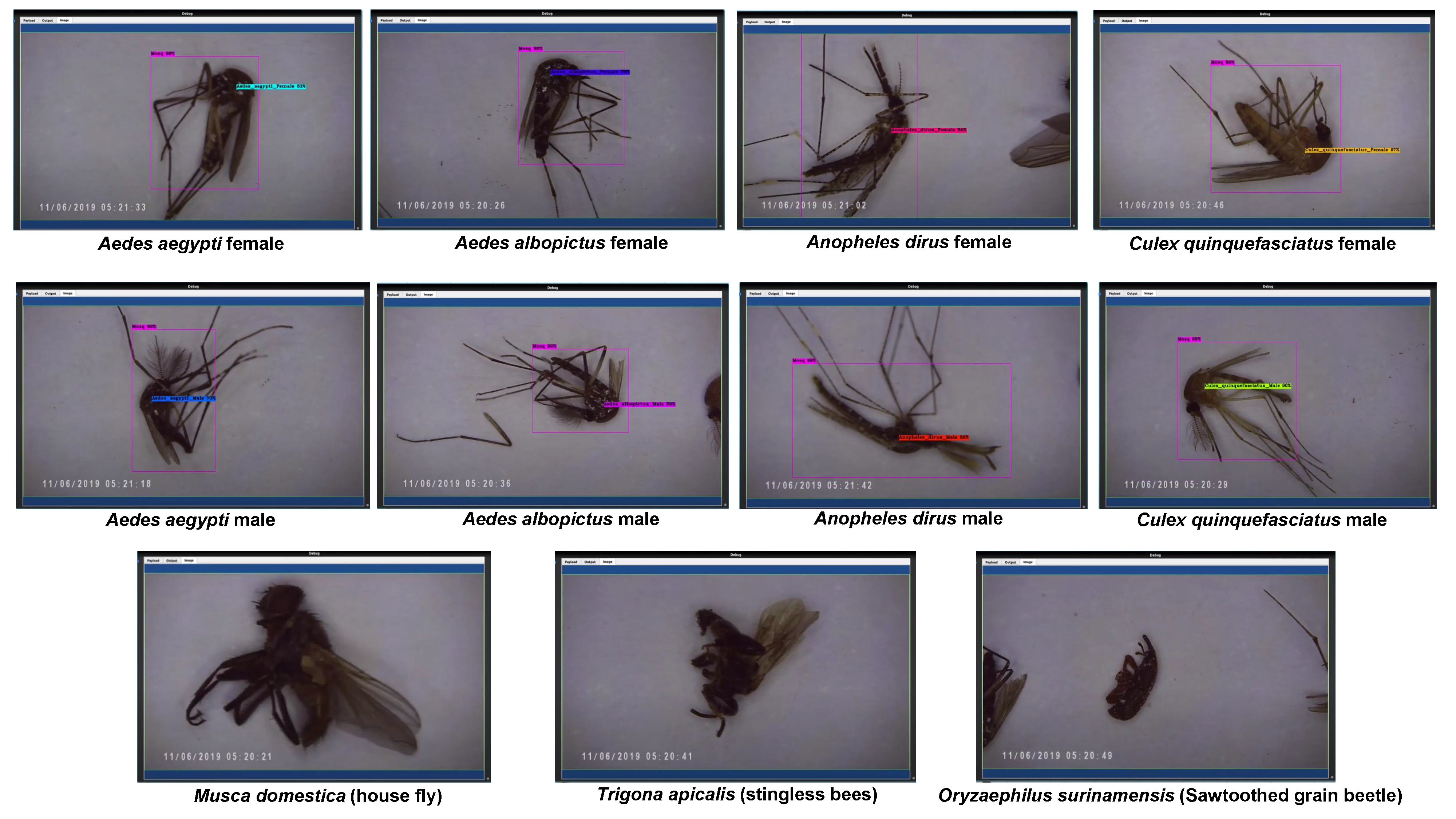

Supplement: Supplementary file 2 — Supplementary Information 2. [file 41598_2021_84219_MOESM2_ESM.tiff]
