# Supplementary material for: Deep learning approaches for challenging species and gender identification of mosquito vectors
Source: Sci Rep. 2021 Mar 1;11:4838. doi: 10.1038/s41598-021-84219-4 (PMC7921658; doi:10.1038/s41598-021-84219-4)
Supplement: Supplementary file 3 — Supplementary Information 3. [file 41598_2021_84219_MOESM3_ESM.docx]

**Deep learning approaches for challenging species and gender identification of mosquito vectors**

**Authors**

Veerayuth Kittichai^1^, Theerakamol Pengsakul^2^, Kemmapon Chumchuen^3^, Yuttana Samang^4^, Patchara Sriwichai^4^, Natthaphop Phatthamolrat^5^, Teerawat Tongloy^5^, Komgrit Jaksukam^5^, Santhad Chuwongin^5^, Siridech Boonsang^5^*

**Supplementary Table S1. Distribution of sorted images dataset.**

| **Class** | **Gender** | **Trained set** | **Testing set** |
| --- | --- | --- | --- |
| *Aedes aegypti* | ♀ | 929 | 228 |
| *Aedes aegypti* | ♂ | 815 | 275 |
| *Aedes albopictus* | ♀ | 702 | 167 |
| *Aedes albopictus* | ♂ | 650 | 177 |
| *Armigeres subalbatus* | ♀ | 461 | 126 |
| *Anopheles dirus* | ♀ | 488 | 192 |
| *Anopheles dirus* | ♂ | 507 | 198 |
| *Culex quinquefasciatus* | ♀ | 626 | 148 |
| *Culex quinquefasciatus* | ♂ | 455 | 166 |
| *Culex spp* | ♂ | 537 | 144 |
| *Culex gelidus* | ♀ | 464 | 135 |
| *Culex vishnui* | ♀ | 251 | 87 |
| *Mansonia annulifera* | ♀ | 309 | 62 |
| *Mansonia uniformis* | ♀ | 255 | 76 |
| *Mansonia indiana* | ♀ | 504 | 128 |
| Non-vector species |  | 212 | 90 |
| **Total** |  | **8,165 (77.29%)** | **2,399 (22.71%)** |

**Supplementary Table S2. Model validation.** The results indicate the quality of the well-trained YOLO v3 model in terms of the number of insect detections during the total use. The model was required to exhibit an accuracy of 100% in the non-vector species identification before it was used to detect the vector species. Liu and colleagues (2020) proposed the following equation to calculate the percentage of confidence: Percentage of Confidence = Pr(Object)× IoU(GT, pred), where Pr (Object) ∈ [0, 1].

| **Vector mosquitoes** | **found / total** | **% confidence** |
| --- | --- | --- |
| *Ae. aegypti* (male & female) | 30/30 | 98-99% |
| *Ae. albopictus* (male & female) | 30/30 | 98-99% |
| *An. dirus* (male & female) | 30/30 | 98-99% |
| *Cu. quinquefasciatus* (male & female) | 30/30 | 98-99% |
| **Non-vector species** |  |  |
| *M. domestica* (house flies) | 30/30 | 98-99% |
| *T. apicalsis* (stingless bees) | 30/30 | 98-99% |
| *O. surinamesis* (sawtroothed grain beetles) | 30/30 | 80-99% |

**Supplementary Table. S3.** Confusion matrix table for class-wise detection. The network model performance of the **YOLO models** indicates the % accuracy of identification for each mosquito species. Generalized accuracy is 78.25 % for one-stage tiny YOLO v2 **(Supporting Table. 3.1)**, 86.14 % for two-stage tiny YOLO v2 **(Supporting Table. 3.2)**, 83.36 % for one-stage YOLO v2 **(Supporting Table. 3.3)**, 87.99 % for two stage YOLO v2 **(Supporting Table. 3.4)**, 86.24% for one-stage YOLO v3 **(Supporting Table. 3.5)** and 88.55% for two-stage YOLO v3 **(Supporting Table. 3.6)** models, respectively. Color intensity each box representatives the % accuracy of the class-wise detection. *Cu. quin* is a representative for *Culex quinquefasciatu*, M for male, and F for female

**Supplementary Table. S3.1** Class-wise detection by using One-stage tiny YOLO v2 model**.**

| Class/ One-stage tiny YOLO v2 | *Ae. aegypti*_F | *Ae. aegypti*_M | *Ae. albopictus*_F | *Ae. albopictus*_M | *An. dirus*_F | *An. dirus*_M | *Ar. subalbatus*_F | *Ma. annulifera*_F | *Ma. uniformis*_F | *Ma. Indiana*_F | *Cu. gelidus*_F | *Cu. quin*_F | *Cu. quin*_M | *Cu. spp*_M | *Cu. vishnui*_F | No.  detected |
| --- | --- | --- | --- | --- | --- | --- | --- | --- | --- | --- | --- | --- | --- | --- | --- | --- |
| *Ae. aegypti*_F | 90.39 | 0.00 | 8.30 | 0.00 | 0.00 | 0.00 | 1.31 | 0.00 | 0.00 | 0 | 0.00 | 0.00 | 0.00 | 0.00 | 0.00 | 229 |
| *Ae. aegypti*_M | 31.84 | 52.24 | 0.00 | 15.92 | 0.00 | 0.00 | 0.00 | 0.00 | 0.00 | 0 | 0.00 | 0.00 | 0.00 | 0.00 | 0.00 | 201 |
| *Ae. albopictus*_F | 0.00 | 0.00 | 92.37 | 7.63 | 0.00 | 0.00 | 0.00 | 0.00 | 0.00 | 0 | 0.00 | 0.00 | 0.00 | 0.00 | 0.00 | 131 |
| *Ae. albopictus*_M | 0.00 | 0.00 | 0.00 | 100.00 | 0.00 | 0.00 | 0.00 | 0.00 | 0.00 | 0 | 0.00 | 0.00 | 0.00 | 0.00 | 0.00 | 167 |
| *An. dirus*_F | 0.00 | 0.00 | 0.00 | 0.00 | 96.88 | 3.12 | 0.00 | 0.00 | 0.00 | 0 | 0.00 | 0.00 | 0.00 | 0.00 | 0.00 | 128 |
| *An. dirus*_M | 0.00 | 0.00 | 0.00 | 0.00 | 0.73 | 99.27 | 0.00 | 0.00 | 0.00 | 0 | 0.00 | 0.00 | 0.00 | 0.00 | 0.00 | 137 |
| *Ar. subalbatus*_F | 0.00 | 0.00 | 0.96 | 0.00 | 0.00 | 0.00 | 98.08 | 0.00 | 0.00 | 0.96 | 0.00 | 0.00 | 0.00 | 0.00 | 0.00 | 104 |
| *Ma. annulifera*_F | 0.00 | 0.00 | 0.00 | 0.00 | 0.00 | 0.00 | 0.00 | 96.30 | 3.7 | 0.00 | 0.00 | 0.00 | 0.00 | 0.00 | 0.00 | 27 |
| *Ma. uniformis*_F | 0.00 | 0.00 | 0.00 | 0.00 | 0.00 | 0.00 | 0.00 | 0.00 | 0.00 | 100.00 | 0.00 | 0.00 | 0.00 | 0.00 | 0.00 | 28 |
| *Ma. Indiana*_F | 0.00 | 0.00 | 0.00 | 0.00 | 0.00 | 0.00 | 0.00 | 0.00 | 0.00 | 100.00 | 0.00 | 0.00 | 0.00 | 0.00 | 0.00 | 84 |
| *Cu. gelidus*_F | 0.00 | 0.00 | 0.00 | 0.00 | 0.00 | 0.00 | 0.00 | 0.00 | 3.13 | 0.00 | 56.25 | 6.25 | 0.00 | 0.00 | 34.38 | 32 |
| *Cu. quin*_F | 0.00 | 0.00 | 0.00 | 0.00 | 0.00 | 0.00 | 0.00 | 0.00 | 0.00 | 1.22 | 0.00 | 97.56 | 0.00 | 1.22 | 0.00 | 82 |
| *Cu. quin*_M | 0.00 | 0.00 | 0.00 | 0.00 | 0.00 | 0.00 | 0.00 | 0.00 | 0.00 | 0.00 | 0.00 | 0.00 | 100.00 | 0.00 | 0.00 | 109 |
| *Cu. spp*_M | 0.00 | 0.00 | 0.00 | 0.00 | 0.00 | 0.00 | 0.00 | 0.00 | 0.00 | 0.00 | 0.00 | 5.51 | 0.00 | 94.49 | 0.00 | 127 |
| *Cu. vishnui*_F | 26.09 | 0.00 | 0.00 | 0.00 | 0.00 | 0.00 | 0.00 | 0.00 | 0.00 | 13.04 | 60.87 | 0.00 | 0.00 | 0.00 | 0.00 | 23 |

**Supplementary Table. S 3.2** Class-wise detection by using Two-stage tiny YOLO v2 model**.**

| Class/ Two-stage tiny YOLO v2 | *Ae. aegypti*_F | *Ae. aegypti*_M | *Ae. albopictus*_F | *Ae. albopictus*_M | *An. dirus*_F | *An. dirus*_M | *Ar. subalbatus*_F | *Ma. annulifera*_F | *Ma. uniformis*_F | *Ma. Indiana*_F | *Cu. gelidus*_F | *Cu. quin*_F | *Cu. quin*_M | *Cu. spp*_M | *Cu. vishnui*_F | No.  detected |
| --- | --- | --- | --- | --- | --- | --- | --- | --- | --- | --- | --- | --- | --- | --- | --- | --- |
| *Ae. aegypti*_F | 90.69 | 0.00 | 8.82 | 0.00 | 0.00 | 0.00 | 0.00 | 0.00 | 0.00 | 0.00 | 0.00 | 0.00 | 0.00 | 0.00 | 0.49 | 204 |
| *Ae. aegypti*_M | 8.14 | 74.66 | 0.00 | 17.19 | 0.00 | 0.00 | 0.00 | 0.00 | 0.00 | 0.00 | 0.00 | 0.00 | 0.00 | 0.00 | 0.00 | 221 |
| *Ae. albopictus*_F | 0.00 | 8.62 | 91.38 | 0.00 | 0.00 | 0.00 | 0.00 | 0.00 | 0.00 | 0.00 | 0.00 | 0.00 | 0.00 | 0.00 | 0.00 | 116 |
| *Ae. albopictus*_M | 0.00 | 0.00 | 0.00 | 98.03 | 0.00 | 0.00 | 0.00 | 0.00 | 0.00 | 0.00 | 0.00 | 0.00 | 0.00 | 0.00 | 0.00 | 152 |
| *An. dirus*_F | 0.00 | 0.00 | 0.00 | 0.00 | 99.09 | 0.91 | 0.00 | 0.00 | 0.00 | 0.00 | 0.00 | 0.00 | 0.00 | 0.00 | 0.00 | 110 |
| *An. dirus*_M | 0.00 | 0.00 | 0.00 | 0.00 | 0.00 | 100.00 | 0.00 | 0.00 | 0.00 | 0.00 | 0.00 | 0.00 | 0.00 | 0.00 | 0.00 | 112 |
| *Ar. subalbatus*_F | 0.00 | 0.00 | 0.00 | 0.00 | 0.00 | 0.00 | 97.98 | 0.00 | 0.00 | 2.02 | 0.00 | 0.00 | 0.00 | 0.00 | 0.00 | 99 |
| *Ma. annulifera*_F | 0.00 | 0.00 | 0.00 | 0.00 | 0.00 | 0.00 | 0.00 | 100.00 | 0.00 | 0.00 | 0.00 | 0.00 | 0.00 | 0.00 | 0.00 | 68 |
| *Ma. uniformis*_F | 0.00 | 0.00 | 0.00 | 0.00 | 0.00 | 0.00 | 0.00 | 5.88 | 23.53 | 70.59 | 0.00 | 0.00 | 0.00 | 0.00 | 0.00 | 17 |
| *Ma. Indiana*_F | 0.00 | 0.00 | 0.00 | 0.00 | 0.00 | 0.00 | 0.00 | 0.00 | 0.86 | 97.41 | 0.00 | 0.86 | 0.00 | 0.00 | 0.00 | 116 |
| *Cu. gelidus*_F | 0.00 | 0.00 | 0.00 | 0.00 | 0.00 | 0.00 | 0.00 | 0.00 | 0.00 | 0.00 | 99.22 | 0.00 | 0.00 | 0.00 | 0.00 | 128 |
| *Cu. quin*_F | 0.00 | 0.00 | 0.00 | 0.00 | 0.00 | 0.00 | 0.00 | 0.00 | 0.00 | 1.32 | 0.66 | 95.36 | 0.00 | 2.65 | 0.00 | 151 |
| *Cu. quin*_M | 0.00 | 0.00 | 0.00 | 0.00 | 0.00 | 0.00 | 0.00 | 0.00 | 0.00 | 0.00 | 0.00 | 0.00 | 90.00 | 10.00 | 0.00 | 150 |
| *Cu. spp*_M | 0.00 | 0.00 | 0.00 | 0.00 | 0.00 | 0.00 | 0.00 | 0.00 | 0.00 | 0.00 | 0.00 | 0.00 | 0.00 | 100.00 | 0.00 | 114 |
| *Cu. vishnui*_F | 0.00 | 0.00 | 0.00 | 0.00 | 0.00 | 0.00 | 0.00 | 0.00 | 0.00 | 0.00 | 63.48 | 1.74 | 0.00 | 0.00 | 34.78 | 115 |

**Supplementary Table. S 3.3** Class-wise detection by using One-stage YOLO v2 model**.**

| Class/ One-stage YOLO v2 | *Ae. aegypti*_F | *Ae. aegypti*_M | *Ae. albopictus*_F | *Ae. albopictus*_M | *An. dirus*_F | *An. dirus*_M | *Ar. subalbatus*_F | *Ma. annulifera*_F | *Ma. uniformis*_F | *Ma. Indiana*_F | *Cu. gelidus*_F | *Cu. quinq*_F | *Cu. quin*_M | *Cu. spp*_M | *Cu. vishnui*_F | No. detected |
| --- | --- | --- | --- | --- | --- | --- | --- | --- | --- | --- | --- | --- | --- | --- | --- | --- |
| *Ae. aegypti*_F | 100.00 | 0.00 | 0.00 | 0.00 | 0.00 | 0.00 | 0.00 | 0.00 | 0.00 | 0.00 | 0.00 | 0.00 | 0.00 | 0.00 | 0.00 | 237 |
| *Ae. aegypti*_M | 8.43 | 91.57 | 0.00 | 0.00 | 0.00 | 0.00 | 0.00 | 0.00 | 0.00 | 0.00 | 0.00 | 0.00 | 0.00 | 0.00 | 0.00 | 261 |
| *Ae. albopictus*_F | 7.90 | 0.00 | 93.22 | 0.00 | 0.00 | 0.00 | 0.00 | 0.00 | 0.00 | 0.00 | 0.00 | 0.00 | 0.00 | 0.00 | 0.00 | 118 |
| *Ae. albopictus*_M | 0.00 | 0.00 | 0.00 | 100.00 | 0.00 | 0.00 | 0.00 | 0.00 | 0.00 | 0.00 | 0.00 | 0.00 | 0.00 | 0.00 | 0.00 | 161 |
| *An. dirus*_F | 0.00 | 0.00 | 0.00 | 0.00 | 93.75 | 6.25 | 0.00 | 0.00 | 0.00 | 0.00 | 0.00 | 0.00 | 0.00 | 0.00 | 0.00 | 64 |
| *An. dirus*_M | 0.00 | 0.00 | 0.00 | 0.00 | 0.00 | 100.00 | 0.00 | 0.00 | 0.00 | 0.00 | 0.00 | 0.00 | 0.00 | 0.00 | 0.00 | 152 |
| *Ar. subalbatus*_F | 0.00 | 0.00 | 0.00 | 0.00 | 0.00 | 0.00 | 100.00 | 0.00 | 0.00 | 0.00 | 0.00 | 0.00 | 0.00 | 0.00 | 0.00 | 19 |
| *Ma. annulifera*_F | 0.00 | 0.00 | 0.00 | 0.00 | 0.00 | 0.00 | 0.00 | 90.00 | 10.00 | 0.00 | 0.00 | 0.00 | 0.00 | 0.00 | 0.00 | 30 |
| *Ma. uniformis*_F | 0.00 | 0.00 | 0.00 | 0.00 | 0.00 | 0.00 | 0.00 | 3.45 | 0.00 | 96.55 | 0.00 | 0.00 | 0.00 | 0.00 | 0.00 | 29 |
| *Ma. Indiana*_F | 0.00 | 0.00 | 0.00 | 0.00 | 0.00 | 0.00 | 0.00 | 0.00 | 0.00 | 100.00 | 0.00 | 0.00 | 0.00 | 0.00 | 0.00 | 39 |
| *Cu. gelidus*_F | 0.00 | 0.00 | 0.00 | 0.00 | 0.00 | 0.00 | 0.00 | 0.00 | 0.00 | 0.00 | 100.00 | 0.00 | 0.00 | 0.00 | 0.00 | 50 |
| *Cu. quin*_F | 0.00 | 0.00 | 0.00 | 0.00 | 0.00 | 0.00 | 0.00 | 0.00 | 0.00 | 0.00 | 0.00 | 90.84 | 0.00 | 9.16 | 0.00 | 131 |
| *Cu. quin*_M | 0.00 | 0.00 | 0.00 | 0.00 | 0.00 | 0.00 | 0.00 | 0.00 | 0.00 | 0.00 | 0.00 | 0.00 | 98.55 | 1.45 | 0.00 | 69 |
| *Cu. spp*_M | 0.00 | 0.00 | 0.00 | 0.00 | 0.00 | 0.00 | 0.00 | 0.00 | 0.00 | 0.00 | 0.00 | 0.00 | 0.00 | 100.00 | 0.00 | 149 |
| *Cu. vishnui*_F | 0.00 | 0.00 | 0.00 | 0.00 | 0.00 | 0.00 | 0.00 | 0.00 | 0.00 | 0.00 | 100.00 | 0.00 | 0.00 | 0.00 | 0.00 | 28 |

**Supplementary Table. S 3.4** Class-wise detection by using Two-stage YOLO v2 model**.**

| Class/ Two-stage YOLO v2 | *Ae. aegypti*_F | *Ae. aegypti*_M | *Ae. albopictus*_F | *Ae. albopictus*_M | *An. dirus*_F | *An. dirus*_M | *Ar. subalbatus*_F | *Ma. annulifera*_F | *Ma. uniformis*_F | *Ma. Indiana*_F | *Cu. gelidus*_F | *Cu. quin*_F | *Cu. quin*_M | *Cu. spp*_M | *Cu. vishnui*_F | No. detection |
| --- | --- | --- | --- | --- | --- | --- | --- | --- | --- | --- | --- | --- | --- | --- | --- | --- |
| *Ae. aegypti*_F | 100.00 | 0.00 | 0.00 | 0.00 | 0.00 | 0.00 | 0.00 | 0.00 | 0.00 | 0.00 | 0.00 | 0.00 | 0.00 | 0.00 | 0.00 | 167 |
| *Ae. aegypti*_M | 4.27 | 93.84 | 0.00 | 1.90 | 0.00 | 0.00 | 0.00 | 0.00 | 0.00 | 0.00 | 0.00 | 0.00 | 0.00 | 0.00 | 0.00 | 211 |
| *Ae. albopictus*_F | 0.00 | 0.00 | 100.00 | 0.00 | 0.00 | 0.00 | 0.00 | 0.00 | 0.00 | 0.00 | 0.00 | 0.00 | 0.00 | 0.00 | 0.00 | 55 |
| *Ae. albopictus*_M | 0.00 | 0.00 | 0.63 | 99.37 | 0.00 | 0.00 | 0.00 | 0.00 | 0.00 | 0.00 | 0.00 | 0.00 | 0.00 | 0.00 | 0.00 | 158 |
| *An. dirus*_F | 0.00 | 0.00 | 0.00 | 0.00 | 98.08 | 1.02 | 0.00 | 0.00 | 0.00 | 0.00 | 0.00 | 0.00 | 0.00 | 0.00 | 0.00 | 52 |
| *An. dirus*_M | 0.00 | 0.00 | 0.00 | 0.00 | 1.00 | 99.20 | 0.00 | 0.00 | 0.00 | 0.00 | 0.00 | 0.00 | 0.00 | 0.00 | 0.00 | 126 |
| *Ar. subalbatus*_F | 0.00 | 0.00 | 0.00 | 0.00 | 0.00 | 0.00 | 100.00 | 0.00 | 0.00 | 0.00 | 0.00 | 0.00 | 0.00 | 0.00 | 0.00 | 50 |
| *Ma. annulifera*_F | 0.00 | 0.00 | 0.00 | 0.00 | 0.00 | 0.00 | 0.00 | 100.00 | 0.00 | 0.00 | 0.00 | 0.00 | 0.00 | 0.00 | 0.00 | 68 |
| *Ma. uniformis*_F | 0.00 | 0.00 | 0.00 | 0.00 | 0.00 | 0.00 | 0.00 | 17.86 | 0.00 | 82.14 | 0.00 | 0.00 | 0.00 | 0.00 | 0.00 | 28 |
| *Ma. Indiana*_F | 0.00 | 0.00 | 0.00 | 0.00 | 0.00 | 0.00 | 0.00 | 0.00 | 1.10 | 73.63 | 0.00 | 23.08 | 0.00 | 0.00 | 2.20 | 91 |
| *Cu. gelidus*_F | 0.00 | 0.00 | 0.00 | 0.00 | 0.00 | 0.00 | 0.00 | 0.00 | 0.00 | 0.00 | 85.37 | 0.00 | 0.00 | 0.00 | 14.63 | 82 |
| *Cu. quin*_F | 0.00 | 0.00 | 0.00 | 0.00 | 0.00 | 0.00 | 0.00 | 0.00 | 0.00 | 0.00 | 0.00 | 100.00 | 0.00 | 0.00 | 0.00 | 155 |
| *Cu. quin*_M | 0.00 | 0.00 | 0.00 | 0.00 | 0.00 | 0.00 | 0.00 | 0.00 | 0.00 | 0.00 | 0.00 | 0.00 | 90.78 | 9.22 | 0.00 | 141 |
| *Cu. spp*_M | 0.00 | 0.00 | 0.00 | 0.00 | 0.00 | 0.00 | 0.00 | 0.00 | 0.00 | 0.00 | 0.00 | 0.00 | 0.00 | 100.00 | 0.00 | 131 |
| *Cu. vishnui*_F | 0.00 | 0.00 | 0.00 | 0.00 | 0.00 | 0.00 | 0.00 | 0.00 | 0.00 | 0.00 | 20.46 | 0.00 | 0.00 | 0.00 | 79.55 | 44 |

**Supplementary Table. S 3.5** Class-wise detection by using One-stage YOLO v3 model**.**

| Class/ One-stage YOLO v3 | *Ae. aegypti*_F | *Ae. aegypti*_M | *Ae. albopictus*_F | *Ae. albopictus*_M | *An. dirus*_F | *An. dirus*_M | *Ar. subalbatus*_F | *Ma. annulifera*_F | *Ma. uniformis*_F | *Ma. Indiana*_F | *Cu. gelidus*_F | *Cu. quin*_F | *Cu. quin*_M | *Cu. spp*_M | *Cu. vishnui*_F | No. detected |
| --- | --- | --- | --- | --- | --- | --- | --- | --- | --- | --- | --- | --- | --- | --- | --- | --- |
| *Ae. aegypti*_F | 100.00 | 0.00 | 0.00 | 0.00 | 0.00 | 0.00 | 0.00 | 0.00 | 0.00 | 0.00 | 0.00 | 0.00 | 0.00 | 0.00 | 0.00 | 188 |
| *Ae. aegypti*_M | 15.79 | 88.55 | 0.00 | 0.00 | 0.00 | 0.00 | 0.00 | 0.00 | 0.00 | 0.00 | 0.00 | 0.00 | 0.66 | 0.00 | 0.00 | 152 |
| *Ae. albopictus*_F | 2.04 | 0.00 | 97.96 | 0.00 | 0.00 | 0.00 | 0.00 | 0.00 | 0.00 | 0.00 | 0.00 | 0.00 | 0.00 | 0.00 | 0.00 | 49 |
| *Ae. albopictus*_M | 0.00 | 0.00 | 1.77 | 98.23 | 0.00 | 0.00 | 0.00 | 0.00 | 0.00 | 0.00 | 0.00 | 0.00 | 0.00 | 0.00 | 0.00 | 58 |
| *An. dirus*_F | 0.00 | 0.00 | 0.00 | 0.00 | 94.60 | 5.40 | 0.00 | 0.00 | 0.00 | 0.00 | 0.00 | 0.00 | 0.00 | 0.00 | 0.00 | 74 |
| *An. dirus*_M | 0.00 | 0.00 | 0.00 | 0.00 | 4.48 | 95.52 | 0.00 | 0.00 | 0.00 | 0.00 | 0.00 | 0.00 | 0.00 | 0.00 | 0.00 | 67 |
| *Ar. subalbatus*_F | 0.00 | 0.00 | 0.00 | 0.00 | 0.00 | 0.00 | 100.00 | 0.00 | 0.00 | 0.00 | 0.00 | 0.00 | 0.00 | 0.00 | 0.00 | 34 |
| *Ma. annulifera*_F | 0.00 | 0.00 | 0.00 | 0.00 | 0.00 | 0.00 | 0.00 | 100.00 | 0.00 | 0.00 | 0.00 | 0.00 | 0.00 | 0.00 | 0.00 | 2 |
| *Ma. uniformis*_F | 0.00 | 0.00 | 0.00 | 0.00 | 0.00 | 0.00 | 0.00 | 0.00 | 25.00 | 75.00 | 0.00 | 0.00 | 0.00 | 0.00 | 0.00 | 12 |
| *Ma. Indiana*_F | 0.00 | 0.00 | 0.00 | 0.00 | 0.00 | 0.00 | 0.00 | 0.00 | 0.00 | 100.00 | 0.00 | 0.00 | 0.00 | 0.00 | 0.00 | 32 |
| *Cu. gelidus*_F | 0.00 | 0.00 | 0.00 | 0.00 | 0.00 | 0.00 | 0.00 | 0.00 | 0.00 | 0.00 | 100.00 | 0.00 | 0.00 | 0.00 | 0.00 | 4 |
| *Cu. quin*_F | 0.00 | 0.00 | 0.00 | 0.00 | 0.00 | 0.00 | 0.00 | 0.00 | 0.00 | 0.00 | 0.00 | 100.00 | 0.00 | 0.00 | 0.00 | 85 |
| *Cu. quin*_M | 0.00 | 0.00 | 0.00 | 0.00 | 0.00 | 0.00 | 0.00 | 0.00 | 0.00 | 0.00 | 0.00 | 0.00 | 98.65 | 1.35 | 0.00 | 74 |
| *Cu. spp*_M | 0.00 | 0.00 | 0.00 | 0.00 | 0.00 | 0.00 | 0.00 | 0.00 | 0.00 | 0.00 | 0.00 | 0.00 | 0.00 | 100.00 | 0.00 | 64 |
| *Cu. vishnui*_F | 0.00 | 0.00 | 0.00 | 0.00 | 0.00 | 0.00 | 0.00 | 0.00 | 0.00 | 0.00 | 100.00 | 0.00 | 0.00 | 0.00 | 0.00 | 3 |

**Supplementary Table. S 3.6** Class-wise detection by using two-stage YOLO v3 model**.**

| Class/ Two-stage YOLO v3 | *Ae. aegypti*_F | *Ae. aegypti*_M | *Ae. albopictus*_F | *Ae. albopictus*_M | *An. dirus*_F | *An. dirus*_M | *Ar. subalbatus*_F | *Ma. annulifera*_F | *Ma. uniformis*_F | *Ma. Indiana*_F | *Cu. gelidus*_F | *Cu. quin*_F | *Cu. quin*_M | *Cu. spp*_M | *Cu. vishnui*_F | No. detected |
| --- | --- | --- | --- | --- | --- | --- | --- | --- | --- | --- | --- | --- | --- | --- | --- | --- |
| *Ae. aegypti*_F | 99.33 | 0.67 | 0.00 | 0.00 | 0.00 | 0.00 | 0.00 | 0.00 | 0.00 | 0.00 | 0.00 | 0.00 | 0.00 | 0.00 | 0.00 | 150 |
| *Ae. aegypti*_M | 0.61 | 98.16 | 0.00 | 1.23 | 0.00 | 0.00 | 0.00 | 0.00 | 0.00 | 0.00 | 0.00 | 0.00 | 0.00 | 0.00 | 0.00 | 163 |
| *Ae. albopictus*_F | 0.00 | 0.00 | 100.00 | 0.00 | 0.00 | 0.00 | 0.00 | 0.00 | 0.00 | 0.00 | 0.00 | 0.00 | 0.00 | 0.00 | 0.00 | 37 |
| *Ae. albopictus*_M | 0.00 | 0.00 | 1.02 | 98.98 | 0.00 | 0.00 | 0.00 | 0.00 | 0.00 | 0.00 | 0.00 | 0.00 | 0.00 | 0.00 | 0.00 | 98 |
| *An. dirus*_F | 0.00 | 0.00 | 0.00 | 0.00 | 100.00 | 0.00 | 0.00 | 0.00 | 0.00 | 0.00 | 0.00 | 0.00 | 0.00 | 0.00 | 0.00 | 68 |
| *An. dirus*_M | 0.00 | 0.00 | 0.00 | 0.00 | 4.29 | 95.71 | 0.00 | 0.00 | 0.00 | 0.00 | 0.00 | 0.00 | 0.00 | 0.00 | 0.00 | 70 |
| *Ar. subalbatus*_F | 3.33 | 0.00 | 0.00 | 0.00 | 0.00 | 0.00 | 96.67 | 0.00 | 0.00 | 0.00 | 0.00 | 0.00 | 0.00 | 0.00 | 0.00 | 30 |
| *Ma. annulifera*_F | 0.00 | 0.00 | 0.00 | 0.00 | 0.00 | 0.00 | 0.00 | 100.00 | 0.00 | 0.00 | 0.00 | 0.00 | 0.00 | 0.00 | 0.00 | 46 |
| *Ma. uniformis*_F | 0.00 | 0.00 | 0.00 | 0.00 | 0.00 | 0.00 | 0.00 | 0.00 | 20.00 | 80.00 | 0.00 | 0.00 | 0.00 | 0.00 | 0.00 | 25 |
| *Ma. Indiana*_F | 0.00 | 0.00 | 0.00 | 0.00 | 0.00 | 0.00 | 0.00 | 0.00 | 0.00 | 94.67 | 0.00 | 4.00 | 0.00 | 0.00 | 1.33 | 75 |
| *Cu. gelidus*_F | 0.00 | 0.00 | 0.00 | 0.00 | 0.00 | 0.00 | 0.00 | 0.00 | 0.00 | 0.00 | 100.00 | 0.00 | 0.00 | 0.00 | 0.00 | 36 |
| *Cu. quin*_F | 0.00 | 0.00 | 0.00 | 0.00 | 0.00 | 0.00 | 0.00 | 0.00 | 0.00 | 0.00 | 0.00 | 99.15 | 0.00 | 0.85 | 0.00 | 118 |
| *Cu. quin*_M | 0.00 | 0.00 | 0.00 | 0.00 | 0.00 | 0.00 | 0.00 | 0.00 | 0.00 | 0.00 | 0.00 | 0.00 | 99.02 | 0.98 | 0.00 | 102 |
| *Cu. spp*_M | 0.00 | 0.00 | 0.00 | 0.00 | 0.00 | 0.00 | 0.00 | 0.00 | 0.00 | 0.00 | 0.00 | 0.00 | 0.00 | 100.00 | 0.00 | 76 |
| *Cu. vishnui*_F | 0.00 | 0.00 | 0.00 | 0.00 | 0.00 | 0.00 | 0.00 | 0.00 | 0.00 | 0.00 | 66.67 | 6.67 | 0.00 | 0.00 | 26.67 | 15 |

**Supplementary Table. S4. Morphological keys to identify the mosquito species.**

| **Species** | **Variability of morphological structure** |
| --- | --- |
| *Aedes aegypti* | 1. Leg. anterior portion of midfemur with a longitudinal white stripe |
|  | 2. Thorax. scutum black or brown with a pair of submedian-longitudinal white stripes |
|  | 3. Thorax. mesepimeron with two well separated white scale patches |
|  | 4. Head. clypeus with white scale patches |
|  | 5. Proboscis without a white band |
| *Aedes albopictus* | 1. Abdomen. abdominal terga with complete basal white bands |
|  | 2. Leg. anterior portion of midfemur without a longitudinal white stripe |
|  | 3. Thorax. scutum with a narrow median-longitudinal white stripe |
|  | 4. Thorax. mesepimeron with white scale patches not separated, forming V-shaped white patch |
|  | 5. Head. clypeus without white scale patches |
|  | 6. Proboscis without sub-median white band |
| *Armigeres subalbatus* | 1. Dorsal head. scales mostly broad and flat |
|  | 2. Proboscis. slightly curved downwards and flattened laterally |
| *Anopheles dirus* | 1. Equal length between palpi and proboscis |
|  | 2. Wing spotted |
| *Culex quinquefasciatus* | 1. Blunt, pale bands on abdominal terga |
|  | 2. Proboscis and tarsi without band |
| *Culex vishnui* | 1. Blunt abdomen |
|  | 2. Leg. dark pale scaled area on hind femur |
| *Culex gelidus* | 1. Proboscis. without accessory pale patches |
|  | 2. Proboscis & tarsi with bands |
| *Mansonia annularis* | 1. Broad, black and pale scales of wings |
|  | 2. Thorax, mesonotum with 4 round white spots and mid-mode of scutellum with broad white scales |
| *Mansonia uniformis* | 1. Broad, black and pale scales of wings |
|  | 2. Thorax, mesonotum marked with a pair of sublateral greenish stripes and scutellum black |
| *Mansonia indiana* | 1. Broad, black and pale scales of wings |
|  | 2. Thorax, mesonotum dark brown with same white scales scutellum black |

**Supplementary Table. S5.** Comparison of performance of neural network models between studies (during year 2019-2020).

| Performance | In this study *^α^* | *Minakshi et al, 2020 ^β^* | *Motta et al, 2020 ^ɤ^* | *Motta et al, 2019* | *Park et al, 2020 ^φ^* | *Couret et al, 2020* *^ε^* |
| --- | --- | --- | --- | --- | --- | --- |
| Recall/ sensitivity (%) | 92.40 | 21 - 87.50 * | 88.97, 99.40 | *NA* | *NA* | *NA* |
| Specificity (%) | 99.40 | *NA* | 98.04, 95.20 | *NA* | *NA* | *NA* |
| Precision (%) | 95.56 | 100 | 89.14, 98.30 | *NA* | *NA* | *NA* |
| Accuracy (%) | 98.9 | *NA* | 93.50, 97.30 | 76.19  [57.14-92.86] | 97.19, 96.86, 90.71 | 96.96, 98.48 |

**(*^α^*)** represent the percentage of each statistical metrics to evaluated the performance of YOLO v3 network, based on IOU = 0.5. Interestingly, the mAP of the YOLO v2 model is 99%.

**(*^β^*)** represent the percentage of each statistical metrics, based on IOU = 0.5, to classify different mosquito parts including thorax, abdomen, wing and leg. (*) represents the percentage of recall varied by their anatomical parts.

***NA*** abbreviates non-applicable.

**(*^ɤ^*)** represents two values of percentage of each statistical metrics, where the first and second values is for species and gender classification, respectively. The study measured the balanced accuracy (BA) to measure the model performance, when the dataset is unbalanced.

**(*^φ^*)** represents the percentage of overall accuracy to measure the performance of VGG16, ResNet50 and SqueezeNet, respectively.

**(*^ε^*)** represents two percentage of overall accuracy, converted from confusion matrix tables, where the first and second values is for species and gender classification, respectively.
